# Meeting the Needs of the People: Fish Consumption Rates in the Pacific Northwest

**DOI:** 10.1289/ehp.121-A334

**Published:** 2013-12-01

**Authors:** Wendee Nicole

**Affiliations:** **Wendee Nicole** was awarded the inaugural Mongabay Prize for Environmental Reporting in 2013. She writes for *Discover*, *Scientific American*, *National Wildlife*, and other magazines.

Native Americans have lived amidst the Pacific Northwest’s pristine rivers and estuaries for millennia, relying on bountiful catches of local fish and shellfish for their sustenance. Because Pacific Northwest tribal populations typically consume much more fish and shellfish than other people in the region,^^1^^ they are exposed to higher levels of toxic chemicals that bioaccumulate in aquatic life—polychlorinated biphenyls, metals, dioxins, and dozens of other toxics found in factory effluent, urban wastewater, and runoff from agriculture and cities.^^2^^^,^^^3^^ As a result, they—along with other groups that eat a lot of fish—face higher risks of developing cancer and other diseases attributable to these chemicals.^^4^^^,^^^5^^

Tribes in Washington, Oregon, and Idaho have joined forces, hoping to lead the way toward cleaner water and safer fish.^^6^^ As Billy Frank, Jr., a Nisqually Tribe member and chairman of the Northwest Indian Fisheries Commission (NWIFC), once wrote, “Fishing defines the tribes as a people. It was the one thing above all else that the tribes wished to retain during treaty negotiations with the federal government 150 years ago. Nothing was more vital to the tribal way of life then, and nothing is more important now.”^^7^^ Even when tribes ceded large portions of their traditional lands and moved to reservations, they insisted on maintaining preexisting rights to harvest aquatic resources, and courts have consistently upheld those rights.

**Figure:**
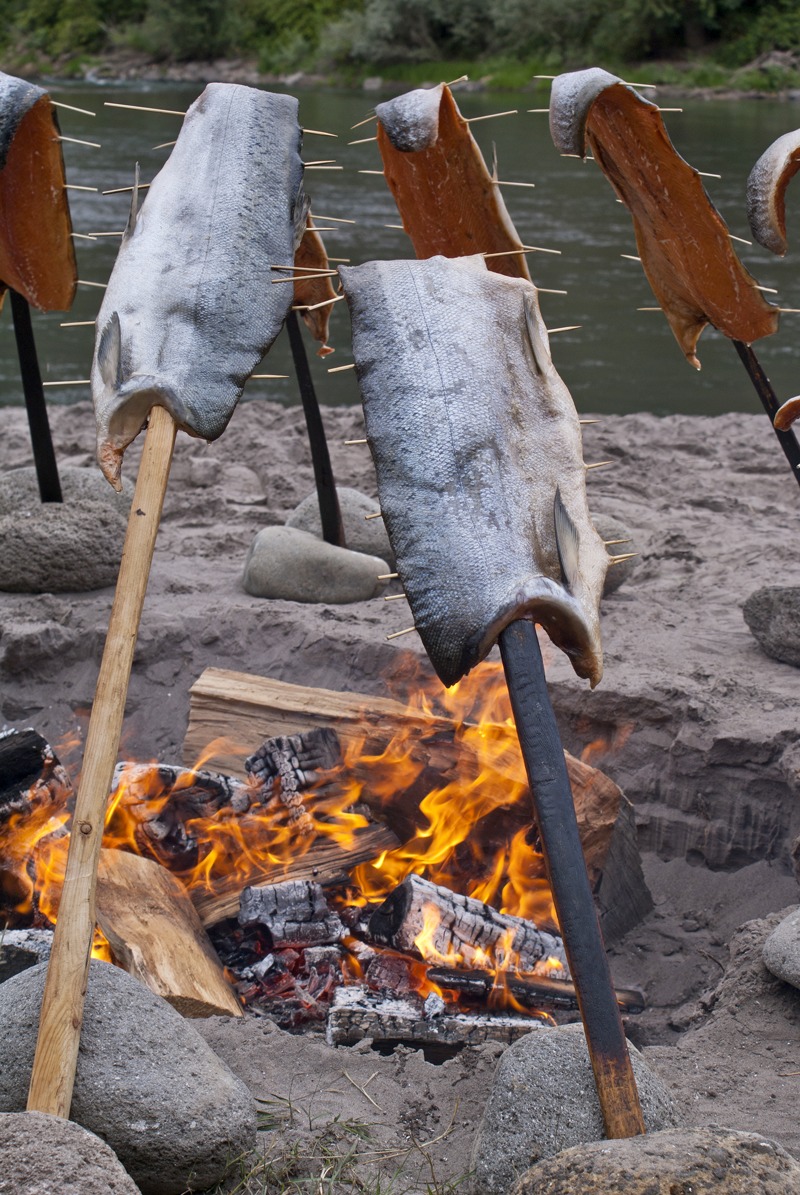
Salmon steaks cook at a festival marking the annual return of the salmon, when the fish travel from the ocean to freshwater streams for spawning season. For the tribes of the Pacific Northwest, seafood plays a spiritual and cultural role in addition to being a dietary staple. © Robert Ice/Getty Images

Pressure from tribal governments led by the Confederated Tribes of the Umatilla Indian Reservation (CTUIR) culminated in Oregon enacting the nation’s most protective state^^8^^ water quality standards in 2011.^^9^^ Now the U.S. Environmental Protection Agency (EPA) would like to see the entire region adopt water quality standards similar to Oregon’s.

“We’re looking for regional consistency,” says Angela Chung, manager of water quality standards for EPA Region 10, which includes Alaska, Idaho, Oregon, Washington, and 271 Indian tribes. Washington and Idaho currently are crafting revised water quality standards, but their processes have become increasingly mired in controversy, as businesses, tribes, politicians, and environmental groups debate how stringent the new standards should be.

## Clean Water Act Redux

More than 40 years have elapsed since the Federal Water Pollution Control Act Amendments of 1972,^^10^^ or Clean Water Act, passed both houses of Congress in a bipartisan manner. President Richard M. Nixon balked at the Act’s $24-billion price tag and vetoed it, but momentum to clean up the nation’s waterways proved strong enough that Congress overrode his veto. Indignant, Nixon impounded half the funds, leading to a 1975 Supreme Court case that ruled “the President cannot frustrate the will of Congress by killing a program through impoundment.”^^11^^

The law’s goal “to restore and maintain the chemical, physical, and biological integrity of the Nation’s waters” included optimistic provisos calling for elimination of all pollution discharges into navigable waterways by 1985 and, in the meantime, “an interim goal of water quality which provides for the protection and propagation of fish, shellfish, and wildlife and provides for recreation in and on the water … by July 1, 1983.”^^12^^ Many refer to this as the Act’s “fishable/swimmable” clause.

The legislation established the National Pollutant Discharge Elimination System (NPDES), which requires entities discharging waste into waterways to obtain permits and obliged states to establish either narrative or numeric water quality standards^^13^^ for both aquatic and human health, which the EPA had to approve.^^14^^

States were slow to enact their own standards, so Congress passed new amendments in 1977 and 1981. Among other things, these amendments required the EPA to develop suggested ambient water quality criteria for 126 priority pollutants as a guide for the states.^^15^^ The criteria represent the highest concentration of each pollutant at which there is not expected to be a significant risk of either cancer or systemic toxicity (i.e., noncancer effects) in humans.^^16^^ These criteria help determine the maximum amount of specific pollutants allowable in effluent.^^14^^

## An End to Delays

Congress grew frustrated over states’ continued hesitancy to enact standards, and in 1987 passed amendments requiring—rather than suggesting—that states adopt numeric water quality standards.^^15^^

By the 1990s, the time for suggestions was over. The EPA issued the National Toxics Rule (NTR) in 1992, putting into place the agency’s recommended water quality criteria for states that hadn’t established their own EPA-approved numeric criteria.^^15^^ While acknowledging that state efforts had been stymied by limited resources and legal challenges, the EPA insisted, “The absence of State water quality standards for toxic pollutants undermines State and EPA toxic control efforts to address these problems. Without clearly established water quality goals, the effectiveness of many of EPA’s water programs is jeopardized.”^^15^^

By the time the NTR completed public review, all but 12 states (Alaska, Arkansas, California, Florida, Idaho, Kansas, Michigan, Nevada, New Jersey, Rhode Island, Vermont, and Washington), plus Puerto Rico and Washington, DC, had adopted EPA-approved human health criteria for water quality standards.^^15^^

One of the variables used to calculate ambient water quality criteria is fish consumption rate, an estimated average of the amount of fish eaten in a given area. Under the NTR, the EPA assumed a fish consumption rate of 6.5 g/day—or one 7-ounce meal per month—but in 2000 the agency recommended that states use a default value of 17.5 g/day, a rate that protects up to the 90th percentile of people in the United States. However, individual states where more fish is eaten should have water quality standards that reflect that, according to EPA guidelines.^^18^^

In the Pacific Northwest, fish consumption can be especially high among tribes as well as recreational anglers, certain minority and immigrant groups, and low-income populations who may ignore fish advisory warnings because they need to put food on the table.^^1^^^,^^^17^^ But despite having populations that eat a lot of fish, the state of Washington still uses the old default value of 6.5 g/day.

Tony Meyer, manager of information services and education for NWIFC, points out this is one of the lowest fish consumption rates in the country. “We’ve been trying to get this rate changed for the past twenty years,” he says.

## A Lightning Rod

Just how wide is the gap between the fish consumption rate used in calculations and the amount Pacific Northwesterners actually eat? At a 2011 meeting between tribes and the Washington state government, the state Department of Ecology (WADOE^^19^^) and NWIFC served thimble-sized pieces of smoked salmon to then-governor Christine Gregoire and others in attendance. Each piece weighed 6.5 g.

But in 2012 WADOE published a document detailing how much fish Washingtonians actually eat.^^1^^ The report reviewed four tribal studies plus data from recreational fishers, and found that the general population averages 19–56 g per day, while tribal members can eat up to 797 g (1.75 pounds) every day.

Michael Grayum, executive director of NWIFC, explained in a September 2012 letter to the EPA Region 10 office that the treaty tribes of western Washington view fish and shellfish as a central component of their spiritual and cultural identity and an important food source. “The longstanding inaccuracies found in the [fish consumption rates] have left tribal communities who rely upon fish and shellfish unduly exposed to toxic chemicals,” he wrote. “This type of environmental policymaking, which provides less protection for a population of people and subsequently leads to the unequal exposure of pollutants, is undoubtedly an environmental injustice.”^^20^^

Dale Norton, manager of WADOE’s Toxics Studies Unit, notes that other factors besides the assumed fish consumption rate affect the calculation of water quality criteria for a pollutant. These include characteristics of the population, bioconcentration factors for toxics, drinking water intake, and relative source contribution (potential exposures from skin absorption, inhalation, and food sources other than fish and water). However, “fish consumption rate has been kind of a lightning rod,” says Norton.

## Searching for Common Ground

WADOE had planned to establish a fish consumption rate above the 6.5-g/day default for its Sediment Management Standards—used to remediate contaminated sites—when industry pushed back, fearing the higher rate would also be used for water quality standards, and concerned about the costs of accommodating it.^^21^^ “Industry was concerned over how the sediment standards would affect surface water quality standards, and they wanted to have a more complete conversation around implementation issues,” says Melissa Gildersleeve, manager of the WADOE Watershed Management Section.

**Figure:**
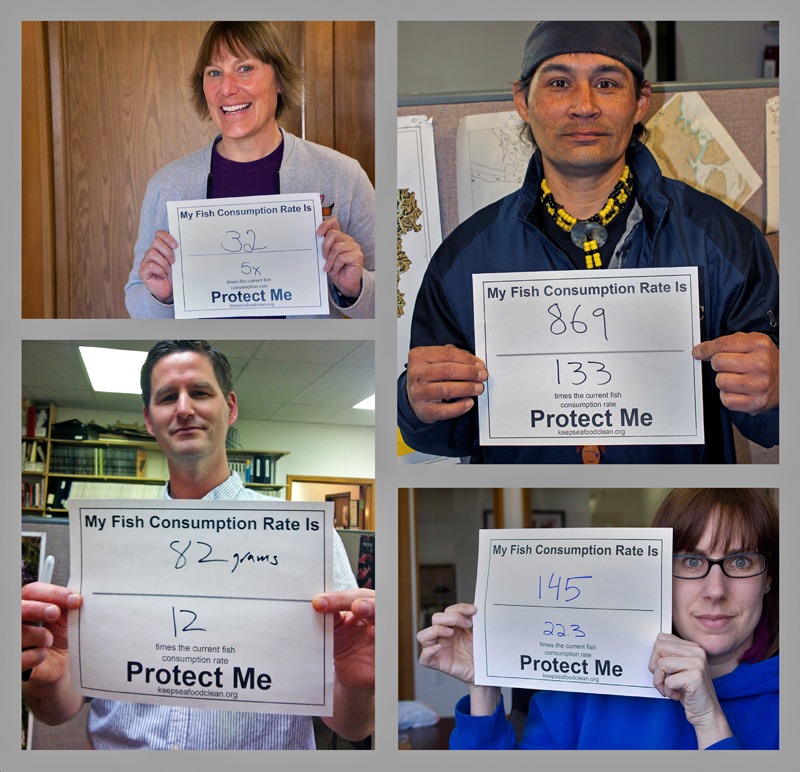
Fish consumption rate is an estimated average of all the fish eaten by a certain population—factoring in both fish eaters and non–fish eaters. In practice, of course, individuals who eat fish often consume much more than this estimated average. These people used the online calculator at http://www.keepseafoodclean.org to compare their personal fish consumption rates against the Washington default value of 6.5 g/day, which works out to one 7-ounce meal per month. © nwifc-photos/Flickr

The Boeing Company, Washington state’s largest employer, reportedly played a large role in the industry response.^^21^^ Joanna Pickup, a spokeswoman for the company, comments, “Boeing’s position is that we support the state’s commitment to find an achievable solution that protects health and the environment and does not negatively impact business operations nor the state’s economy.”

The pushback worked. In July 2012 WADOE changed course, announcing a narrative standard for Washington’s Sediment Management Standards rule.^^22^^ Additionally, no statewide fish consumption rate would be named; instead, rates would be determined on a site-by-site basis. The narrative standards adopted in the Sediment Management Standards rule require the state to consider tribal fish consumption rates when establishing sediment cleanup standards.

Environmental groups and tribes were disappointed; they had, in fact, hoped that once WADOE determined a fish consumption rate for the sediment rule, it would guide the water quality standards, up next for review. Instead, WADOE implemented a public process called the Delegates’ Table, in which tribes, environmental groups, industry, and the interested public would participate in a statewide stakeholder process to develop water quality standards. In frustration, all the environmental groups and almost all the tribes withdrew or refused to participate.^^23^^^,^^^24^^

“The tribes are sitting out of that process, and for good reason,” says Meyer. “We thought we were moving forward this last year, and [WADOE] slowed the process, and they turned it into a stakeholder process. Tribes aren’t stakeholders. Stakeholders are like industry and environmental groups. Tribes are governments, and they need to be dealt with as such.”

The tribes invoked the federal Indian trust responsibility, which, as the Bureau of Indian Affairs explains it, is “a legally enforceable fiduciary obligation on the part of the United States to protect tribal treaty rights, lands, assets, and resources, as well as a duty to carry out the mandates of federal law.”^^25^^ They opted for a government-to-government approach and have had meetings with EPA Region 10. “They came to us and said, ‘In your tribal trust capacity as our federal partner, we would like to just start meeting with you to discuss our concerns, because at the end of the day you have to approve or disapprove what the state submits,’ ” says the EPA’s Chung.

**Figure:**
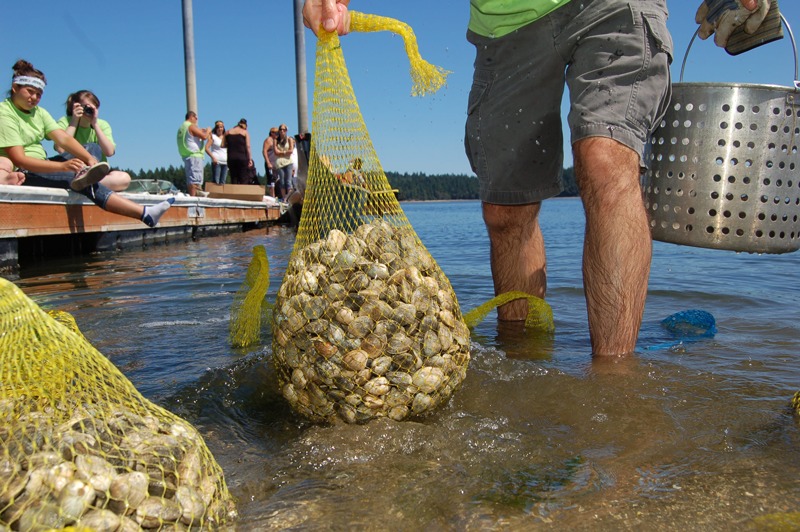
The Squaxin Island Tribe of Washington honors the first salmon caught of the year with a seafood festival. A 1994 survey of Squaxin Island tribal members found median fish consumption rates of 66 g/day for men and 25 g/day for women.^37^ © nwifc-photos/Flickr

The EPA has authority under the Clean Water Act to step in and set revised water quality standards, and the agency reminded WADOE of this in June 2013. “The best available science includes evidence of consumption well above 6.5 g/day among high fish consumers and shows that the human health criteria currently in effect … are not sufficiently protective,” wrote Region 10 administrator Dennis J. McLerran. “Should Washington’s process be unnecessarily delayed, the EPA has the authority to amend the NTR human health criteria for Washington.”^^26^^

The state has defined a timeline for revising the standard “and has been involving and communicating with a number of interested parties in a very public process,” says Chung. “We think that’s a very important piece to developing standards that will last and won’t be immediately challenged.” The EPA indicated it likely will not intervene if Washington follows its established schedule.^^26^^

## More Frustration

But despite WADOE’s work with industry, tribes, and environmentalists to develop the new standards and implementation tools, four environmental groups and two commercial fishing organizations^^27^^ sent a 60-day intent-to-sue notice to the EPA in July 2013 for not enforcing the Clean Water Act in Washington state.^^28^^ The groups requested immediate action by the EPA to, at minimum, establish a fish consumption rate so they can move on to discussing implementation of new standards—which will be challenging in itself.

However, the groups are not enthusiastic about the implementation ideas that WADOE has floated so far. “There are talks of compliance schedules several decades long. There’s simply no precedence under the Clean Water Act for variances [that persist over] multiple permit cycles,” says Bart Mihailovich, director of Spokane Riverkeeper, one of the groups that sent the notice. A typical variance—an allowance granted by the EPA for a facility to not meet water quality standards—lasts the duration of a single five-year permit cycle.

“I don’t consider what we’re doing as loopholes,” says WADOE’s Gildersleeve. “We’re requiring businesses to put together pollution plans that they would implement in their NPDES permits, and they would have to take actions to reduce toxics. If it looks like they’re not able to meet [the standards] because the technology doesn’t exist, then we’ll consider looking at a variance. They would have up to forty years for some chemicals like PCBs that are pervasive in the environment.” She acknowledges that the EPA may not approve this idea.

“We recognize that new permit limits will be challenging for industry,” says Sandy Howard, WADOE communications manager. “We feel we need to do this to protect current and future generations. If we don’t update our standards now, EPA or the courts will do it for us.”

## Oregon’s Experience

Although Washington differs in several ways from its southern neighbor—including having a larger population,^^29^^^,^^^30^^ a larger number of federally recognized tribes,^^31^^ more industry,^^32^^ and two coastlines (the Pacific Ocean and Puget Sound) instead of one—WADOE has looked to Oregon to learn from its success: The water quality standards rulemaking process there won Oregon’s Department of Environmental Quality (ORDEQ), the EPA, and CTUIR, plus a facilitator, awards from the U.S. Institute for Environmental Conflict Resolution.^^33^^

“It was rocky in the beginning, and in the end I attribute a lot of the success to the tribe’s leadership,” says Andrea Matzke, a water quality specialist with ORDEQ. “The three governments [federal, state, and tribal] collaborated, and that was instrumental.”

In 2004 Oregon’s Environmental Quality Commission had adopted a 17.5-g/day fish consumption rate, as per the EPA’s nationwide suggested guideline. “The tribes, particularly, had a lot of concerns and were saying 17.5 doesn’t really represent what many of the tribal community eat here, and so they talked a lot with EPA, and [EPA] delayed their approval,” explains Matzke.

Considering the real possibility that the EPA might reject its submitted standards, ORDEQ decided to work closely with the tribes and the EPA to develop new standards through a public process.^^34^^ The state came to an agreement with the tribes and the EPA to use a 175-g/day fish consumption rate, which protects up to the 95th percentile of Oregonians who consume the most fish, according to research conducted by the Columbia River Inter-Tribal Fish Commission, the EPA, and tribal biologists.^^35^^^,^^^36^^^,^^^37^^^,^^^38^^^,^^^39^^ The revised human health criteria were approved by the Oregon Environmental Quality Commission in 2011^^9^^ and by the EPA a few months later.^^40^^

The state also worked with tribes and industry on implementation tools to enable factories and municipalities to comply with the stricter standards. While the tribes made concessions (Carl Merkle, a salmon recovery policy analyst for the CTUIR, says many tribal members wanted an even higher rate than 175 g/day), the result was something all parties could live with. Merkle points out that the state reached the compromise “sometimes at great institutional and political cost.” He says, “We benefited from some state officials in Oregon who showed remarkable courage.”

**“**It wasn’t a slam dunk. It was really hard work,” says Mary Lou Soscia, Columbia River coordinator for EPA Region 10.

Oregon’s new rate raised the bar for the region, and the EPA has repeatedly told tribes it would like to see similar rates in Washington and Idaho.^^41^^ Alaskan officials indicated they may soon revise that state’s standards, and Idaho is in the process now.

Idaho updated its standards in 2006 using a fish consumption rate of 17.5 g/day, but the EPA did not act to approve or disapprove the standards until 2012, when a lawsuit from environmental groups upped the pressure. At that point “we disapproved the Idaho rate because they hadn’t done an adequate review of existing fish consumption data out there,” says Soscia. Idaho is back at the drawing board.

## Do Standards Make a Difference?

Don Essig, water quality standards coordinator for Idaho DEQ, remains skeptical that tighter standards are the most efficient way to reduce toxics. “The lower criteria aren’t really going to get to what people want, which is less contamination,” he says. “It will create a whole lot more waterways that are listed as impaired and will create a lot of work for the department … that I don’t think in the end is really going to change things on the landscape.”

Jennifer Wigal, water quality standards and assessments manager for ORDEQ, agrees that “the level of significance the standards have for any particular situation is going to vary.” Legacy toxics such as polychlorinated biphenyls, which remain in the environment decades after they were banned, are particularly hard to clean up and manage.

**Figure:**
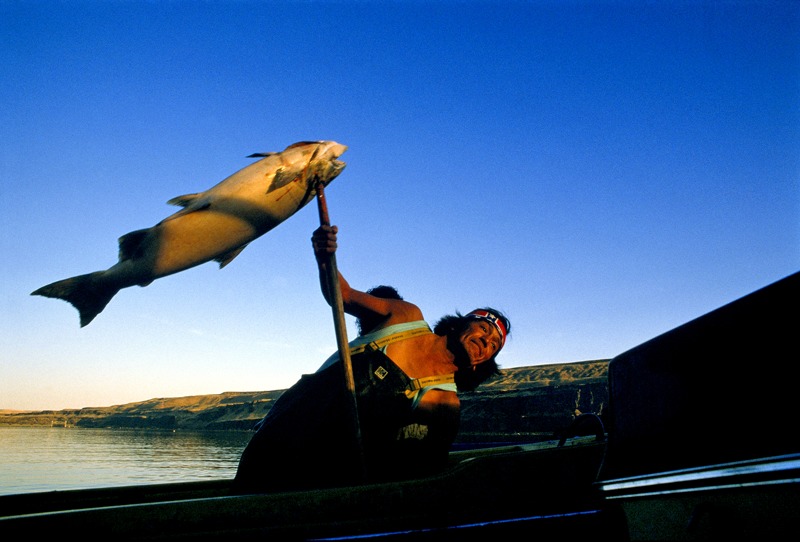
A Yakima Indian hauls a king salmon into his boat in Washington. The regulation of pollutants involves tough questions about what’s feasible and what’s achievable. At the same time, says Chris Wilke of Puget Sound Waterkeepers, “We’re talking about people’s health here, not some theoretical environmental protection for one sensitive species. In this case, the sensitive species is people.” © Natalie Fobes/Getty Images

“All the pollutants have their own story,” Wigal says. “Some have natural sources. Some are purely manmade. Some you don’t find often, and some are ubiquitous. [For ubiquitous toxics], standards aren’t going to be the end-all-be-all to reduce the levels of those pollutants in the water. A lot of other things need to happen, whether it’s cleanup programs or air programs or consumer choices or preferences.”

Soscia disagrees with Essig that tighter standards won’t make much difference on the ground. “EPA would expect that more stringent criteria in NPDES permits will result in reduced toxic discharges and a long-term reduction in toxics in the environment over time,” she says. “We have a lot of toxics problems in our rivers and streams, and … at the highest level, EPA is committed to ensuring protection for high fish consumers.” In the end, though, Essig says, tough standards are meaningless without enforcement. And there will always be some level of risk.

“The dialog that I hear is that what we have now is unfair because some people have higher risk,” Essig says. “That will always be the case; risks are inherently unequal. We can shift the level of protection higher, for everyone, and yes, that is good, but it comes at a cost.” He adds, “If there were no tradeoffs [or] costs involved, the answer would be quite simple—go for the highest of the high.”

If the EPA wants to see the entire Pacific Northwest region with fish consumption rates in the ballpark of 175 g/day, will other U.S. regions soon follow? Not including tribal lands, the state with the next highest fish consumption rate used to develop water quality standards is Maine at 32.2 g/day, followed by Minnesota and Alabama at 30 g/day. All other states fall in the teens or lower.^^42^^ Ten other states besides Washington still use the original rate of 6.5 g/day, although several of these states are currently updating their water quality standards.

Florida is set to revise its standards in what may prove to be a contentious battle, if Washington’s experience is any indication.^^43^^^,^^^44^^ The EPA just released a study of recreational fishing in Florida, Connecticut, Minnesota, and North Dakota, revealing substantially higher fish consumption than the default value of 17.5 g/day.^^45^^

## Getting to Implementation

Although implementing new standards costs money, Chris Wilke, director of Puget Sound Waterkeepers, underscores that cleaner water provides economic benefits.^^46^^^,^^^47^^^,^^^48^^ “Commercial fishing used to be very strong in Washington state. We used to have a lot of canneries in Puget Sound, and we have a suppressed consumption rate^^1^^ because there are not as many fish as there used to be,” he says.

If waters get cleaned up and fish populations rebound, Wilke reasons, recreational and commercial fishing could expand. Tightening water quality standards also creates benefits in terms of avoided health costs for diseases attributable to toxic pollutants in water.^^47^^^^,49^^

“Fishable/swimmable water is guaranteed to all citizens under the Clean Water Act, and if it’s not clean, it needs to be cleaned up,” Wilke says. “So often, environmental issues get distilled down into jobs versus the environment, and we’re talking about people’s health here, not some theoretical environmental protection for one sensitive species. In this case, the sensitive species is people.”

Wilke gives an example of how, in practice, current standards fail to protect anglers. “Currently we have a fish consumption advisory in Puget Sound for resident Chinook [blackmouth] because of PCBs,” he says. The Department of Health recommends no more than two blackmouth meals per month. “One fish weighs up to twelve pounds, and yet the Department of Fish and Wildlife gives people twenty punches on their card,” he says, referring to the mandatory Catch Record Cards that anglers use to track the number and species of fish they land. In other words, it could be argued that there is implicit encouragement to catch more fish than may be safe to eat—avid blackmouth anglers likely consume far more than two meals per month. A disconnect exists between the health advisory and the fishing regulations.

“We all deserve to be able to eat fish without the fear that it will make us sick,” says Mihailovich, Indeed, the right to fishable/swimmable water was afforded to all citizens through the 1972 Clean Water Act, although it has taken several decades to see its provisions fully realized.

“The time to study this is over,” Mihailovich says. “There are so many studies out there that show we are off target [regulation-wise], and the current number doesn’t protect us. We need to look at implementation.”
